# Measurement of Exercise Tolerance before Surgery (METS) study: a protocol for an international multicentre prospective cohort study of cardiopulmonary exercise testing prior to major non-cardiac surgery

**DOI:** 10.1136/bmjopen-2015-010359

**Published:** 2016-03-11

**Authors:** Duminda N Wijeysundera, Rupert M Pearse, Mark A Shulman, Tom E F Abbott, Elizabeth Torres, Bernard L Croal, John T Granton, Kevin E Thorpe, Michael P W Grocott, Catherine Farrington, Paul S Myles, Brian H Cuthbertson

**Affiliations:** 1 St. Michael's Hospital/Toronto General Hospital/University of Toronto, Toronto, Ontario, Canada; 2 Queen Mary University of London, London, UK; 3 Alfred Hospital/Monash University, Melbourne, Victoria, Australia; 4 St. Michael's Hospital, Toronto, Ontario, Canada; 5 NHS Grampian, Aberdeen, UK; 6 University Health Network/Mount Sinai Hospital/University of Toronto, Toronto, Ontario, Canada; 7 University of Toronto/St. Michael's Hospital, Toronto, Ontario, Canada; 8 University Hospital Southampton/University of Southampton, Southampton, UK; 9 Sunnybrook Health Sciences Centre/University of Toronto, Toronto, Ontario, Canada

**Keywords:** Preoperative assessment, Cardiopulmonary exercise testing, Risk prediction, Postoperative complications, Natriuretic peptides

## Abstract

**Introduction:**

Preoperative functional capacity is considered an important risk factor for cardiovascular and other complications of major non-cardiac surgery. Nonetheless, the usual approach for estimating preoperative functional capacity, namely doctors’ subjective assessment, may not accurately predict postoperative morbidity or mortality. 3 possible alternatives are cardiopulmonary exercise testing; the Duke Activity Status Index, a standardised questionnaire for estimating functional capacity; and the serum concentration of *N*-terminal pro-B-type natriuretic peptide (NT pro-BNP), a biomarker for heart failure and cardiac ischaemia.

**Methods and analysis:**

The Measurement of Exercise Tolerance before Surgery (METS) Study is a multicentre prospective cohort study of patients undergoing major elective non-cardiac surgery at 25 participating study sites in Australia, Canada, New Zealand and the UK. We aim to recruit 1723 participants. Prior to surgery, participants undergo symptom-limited cardiopulmonary exercise testing on a cycle ergometer, complete the Duke Activity Status Index questionnaire, undergo blood sampling to measure serum NT pro-BNP concentration and have their functional capacity subjectively assessed by their responsible doctors. Participants are followed for 1 year after surgery to assess vital status, postoperative complications and general health utilities. The primary outcome is all-cause death or non-fatal myocardial infarction within 30 days after surgery, and the secondary outcome is all-cause death within 1 year after surgery. Both receiver-operating-characteristic curve methods and risk reclassification table methods will be used to compare the prognostic accuracy of preoperative subjective assessment, peak oxygen consumption during cardiopulmonary exercise testing, Duke Activity Status Index scores and serum NT pro-BNP concentration.

**Ethics and dissemination:**

The METS Study has received research ethics board approval at all sites. Participant recruitment began in March 2013, and 1-year follow-up is expected to finish in 2016. Publication of the results of the METS Study is anticipated to occur in 2017.

**Strengths and limitations of this study**

- A large generalisable sample of 1723 participants at multiple centres worldwide will be used to estimate the prognostic accuracy of cardiopulmonary exercise testing, the Duke Activity Status Index and the serum concentration of N-terminal pro-B-type natriuretic peptide.
- The study involves detailed prospective follow-up after surgery to ascertain survival, major complications and general health utilities.
- Participants, healthcare personnel and outcome adjudicators are blinded to cardiopulmonary exercise testing results, Duke Activity Status Index scores and serum N-terminal pro-B-type natriuretic peptide concentration, thereby facilitating unbiased estimates of their prognostic accuracy.
- An important potential limitation is selection bias introduced by individuals who meet eligibility criteria, are theoretically capable of exercising, but decline to participate in a research study of exercise testing. Such non-participants may be systematically different due to possible higher likelihood of having other markers of poor health (eg, smoking).

## Introduction

More than 300 million individuals undergo major surgery worldwide every year, and many are at risk for postoperative cardiovascular complications.^1^ ^2^ Clinical practice guidelines recommend preoperative risk stratification as a component of any strategy to prevent these complications.^3^ Risk-stratification algorithms proposed by several international guidelines emphasise the assessment of preoperative fitness or functional capacity.^3^ ^4^ For example, the current American College of Cardiology and American Heart Association guidelines recommend that patients be allowed to proceed directly to elective major non-cardiac surgery if they are deemed capable of more than four metabolic equivalents of activity without symptoms.^3^ Preoperative functional capacity is also a versatile measure of perioperative risk since it may stratify risk for non-cardiovascular complications such as pneumonia, respiratory failure and infection.^5–9^

The current standard of care for assessing preoperative functional capacity involves a doctor making a subjective estimate after interviewing the patient. Previous studies highlight potential limitations with this approach, including poor accuracy when predicting death or complications after non-cardiac surgery,^10^ ^11^ as well as poor agreement with validated measures of functional capacity.^12^ These limitations point to the need for more accurate alternatives to assess preoperative functional capacity and, in turn, surgical outcomes. Three potential options are cardiopulmonary exercise testing (CPET), which is often considered to be the ‘gold standard’ non-invasive assessment of functional capacity; the Duke Activity Status Index (DASI),^13^ which is a standardised questionnaire with demonstrated correlation to gold standard measures of functional capacity; and the serum concentration of *N*-terminal pro-B-type natriuretic peptide (NT pro-BNP), which is biomarker for heart failure or cardiac ischaemia.

CPET requires patients to undergo symptom-limited incremental exercise on a bicycle or treadmill for 8–12 min while undergoing continuous spirometry. Indices of cardiorespiratory performance are simultaneously measured, with the most common being peak oxygen consumption (VO_2_ peak) and anaerobic threshold (AT). Recent systematic reviews and individual studies largely support preoperative CPET as a predictor of complications after surgery,^14–16^ but acknowledge important limitations. For example, many prior studies have important methodological problems. Specifically, very few studies blinded caregivers or outcome adjudicators to CPET results,^17–19^ thereby potentially biasing estimates of prognostic accuracy in the vast majority of previous studies.^20^ In addition, many studies have limited generalisability due to small sample sizes and single-centre designs. Thus, despite the theoretical promise of CPET in the perioperative setting, higher quality evidence remains needed to confirm its prognostic accuracy, identify patients who warrant this expensive and specialised test, and provide a robust argument for its wider implementation.

The DASI is a 12-item self-administered questionnaire enquiring about activities of daily living. It has construct and criterion validity as a measure of functional capacity in surgical patients.^21^ ^22^ No large study has evaluated the prognostic accuracy of a preoperative DASI score for predicting outcomes after surgery.

While no blood test can quantify functional capacity, serum concentration of NT pro-BNP may indirectly fulfil this role by serving as an integrated marker of cardiac dysfunction, including myocardial stretch and ischaemia.^23^ ^24^ Emerging data, which include several individual studies from our group as well as meta-analyses,^25–29^ have found preoperative NT pro-BNP concentrations to have reasonable prognostic accuracy in predicting death and cardiac complications after non-cardiac surgery.

To help develop improved methods to measure preoperative functional capacity and incorporate it into overall surgical risk assessment, we are conducting the Measurement of Exercise Tolerance before Surgery (METS) Study. The main objectives of this multicentre prospective cohort study are presented below.

### Primary objective

- To compare preoperative CPET to subjective assessment for predicting death or non-fatal myocardial infarction (MI) within 30 days after major elective non-cardiac surgery.

### Secondary objectives

1. To compare CPET to subjective assessment for predicting death within 1 year after major elective non-cardiac surgery.
2. To compare preoperative DASI, NT pro-BNP, CPET and subjective assessment for predicting death or non-fatal MI within 30 days after non-cardiac surgery.
3. To compare preoperative DASI, NT pro-BNP, CPET and subjective assessment for predicting death within 1 year after major elective non-cardiac surgery.

## Methods and analysis

### Study design

**Figure 1:**
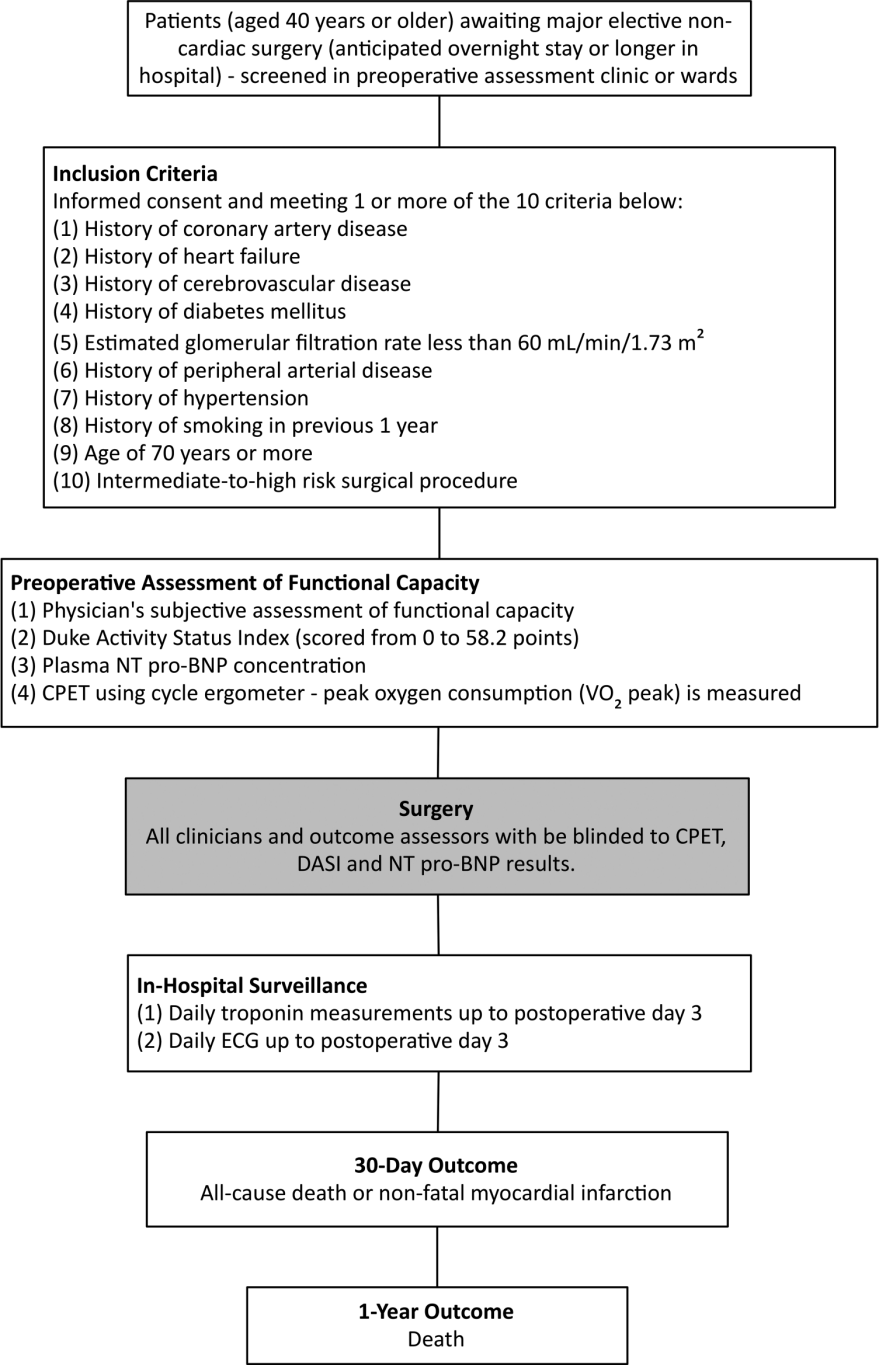
Overall design of the METS Study. CPET, cardiopulmonary exercise test; DASI, Duke Activity Status Index; METS, Measurement of Exercise Tolerance before Surgery; NT pro-BNP, N-terminal pro-B-type natriuretic peptide; VO_2_, oxygen consumption.

The METS Study is a multinational prospective cohort study of 1723 patients undergoing major elective non-cardiac surgery at participating centres in Australia, Canada, New Zealand and the UK. The overall study design is outlined in figure 1.

### Participant eligibility criteria

Potential participants are recruited from the preoperative assessment clinics or surgical wards of participating sites. To be eligible to participate in the METS Study, individuals must be aged 40 years or older, and scheduled to undergo elective non-cardiac surgery under general and/or regional anaesthesia with a minimum of an overnight hospital stay for medical reasons. In addition, they must have one or more clinical risk factors for perioperative cardiac complications or coronary artery disease (table 1). Exclusion criteria are presented on box 1 and table 2. All participants provide informed consent at time of recruitment to the study.

Box 1 **Exclusion criteria for the Measurement of Exercise Tolerance before Surgery (METS) Study**

- At the time of approach for potential recruitment to study, inadequate time to feasible complete cardiopulmonary exercise testing (CPET) before surgery (defined as less than 24 h)
- Planned use of CPET for preoperative risk stratification independent of METS study protocol
- Planned surgery exclusively performed by an endovascular approach (eg, endovascular aortic aneurysm repair)
- Presence of an automated implantable cardioverter-defibrillator
- Known or suspected pregnancy
- Previous enrolment in the METS Study
- Active cardiac conditions,^59^ absolute contraindications to CPET (American Thoracic Society and American College of Chest Physicians guidelines)^30^ and conditions expected to preclude CPET (eg, lower limb amputation, severe claudication)
- Systolic blood pressure ≥180 mm Hg and diastolic blood pressure ≥100 mm Hg at the time of potential study recruitment

**Table 1:** Clinical risk factors required for inclusion in the METS Study*

Risk factor	Definition
Intermediate-to-high risk surgery	Intraperitoneal, intrathoracic or major vascular (suprainguinal or lower extremity vascular) procedures
Coronary artery disease	History of angina; myocardial infarction; positive exercise, nuclear or echocardiographic stress test; resting wall motion abnormalities on echocardiogram; coronary angiography with evidence of ≥50% vessel stenosis; or ECG with pathological Q-waves in two contiguous leads
Heart failure	History of heart failure or diagnostic chest X-ray (ie, pulmonary vascular redistribution or pulmonary oedema)
Cerebrovascular disease	History of stroke or transient ischaemic attack; or imaging (CT or MRI) evidence of previous stroke
Diabetes mellitus	Requirement for insulin or oral hypoglycaemic therapy
Preoperative renal insufficiency	Requirement for renal replacement therapy before surgery, or estimated glomerular filtration rate† less than 60 mL/min/1.73 m^2^
Peripheral arterial disease	History of peripheral arterial disease; ischaemic intermittent claudication; rest pain; lower limb revascularisation procedure; peripheral arterial obstruction of ≥50% luminal diameter; or resting ankle/arm systolic blood pressure ratio ≤0.90
Hypertension	Physician diagnosis of hypertension
Smoker	History of smoking within 1 year before surgery
Advanced age	70 years or older

*One or more of these risk factors must be present to meet the study eligibility criteria.

†Estimated using the MDRD Study equation.^58^

MDRD, Modification of Diet in Renal Disease; METS, Measurement of Exercise Tolerance before Surgery.

**Table 2:** Definitions of specific exclusion criteria in the METS Study

Active cardiac conditions^59^	Acute coronary syndrome: myocardial infarction within prior 30 days, unstable angina, or severe angina (Canadian Cardiovascular Society class III or IV)
	Decompensated heart failure (New York Heart Association functional Class IV), new onset heart failure, or worsening heart failure
	Significant arrhythmias: atrioventricular heart block (high grade, Mobitz II, third-degree); symptomatic ventricular arrhythmias; supraventricular arrhythmias with uncontrolled ventricular rate (ie, >100 bpm at rest); symptomatic bradycardia; or newly recognised ventricular tachycardia
	Severe valvular disease: severe aortic stenosis (mean pressure gradient >40 mm Hg, aortic valve area <1.0 cm^2^ or symptomatic aortic stenosis); or symptomatic mitral stenosis (progressive dyspnoea on exertion, exertional presyncope or heart failure)
Absolute contraindications to CPET^30^	Recent acute myocardial infarction (3–5 days) or unstable angina
	Uncontrolled arrhythmias causing symptoms or haemodynamic compromise
	Syncope
	Active endocarditis
	Acute myocarditis or pericarditis
	Symptomatic severe aortic stenosis
	Uncontrolled heart failure or pulmonary oedema
	Acute pulmonary embolus or pulmonary infarction
	Thrombosis of lower extremities
	Suspected dissecting aneurysm
	Uncontrolled asthma or respiratory failure
	Oxygen saturation at rest less than 85%
	Acute non-cardiopulmonary disorder that may affect exercise performance or be aggravated by exercise (ie, infection, renal failure, thyrotoxicosis)
	Mental impairment leading to inability to cooperate

CPET, cardiopulmonary exercise testing; METS, Measurement of Exercise Tolerance before Surgery.

### Preoperative cardiopulmonary exercise testing

During the period from study recruitment to 1 day before surgery, participants undergo symptom-limited incremental CPET on a computer-controlled, electromagnetically braked cycle ergometer, under physician supervision and in accordance with published guidelines.^30^ Prior to CPET, each participant performs spirometry with forced inspiratory and expiratory flow volume loops. The subsequent incremental exercise test takes 8–12 min to complete. It follows a preliminary 3 min resting period, during which the participant sits on the cycle ergometer while cardiovascular and respiratory measurements are taken, and 3 min of unloaded cycling (0 W) that serves a warm up. At testing sites where the cycle ergometers cannot be set to 0 W, the unloaded cycling phase is set at the minimum workload possible on the local cycle ergometer. Pedalling resistance is then increased progressively every minute using a ramped protocol during which participants pedal at 60 revolutions per minute. Typically, work rates are increased by 10 W per minute in untrained individuals, and by up to 20–30 W per minute in well-trained participants or those that participate regularly in physical activity.

Participants exercise until they reach their limit of tolerance (ie, unable to pedal at 60 revolutions per minute despite encouragement), stop for non-cardiopulmonary reasons or are instructed to stop based on safety-based termination criteria.^30^ Reasons for termination are documented for all tests. Participants undergo breath-by-breath measurement of minute ventilation, oxygen uptake and carbon dioxide production from expired gas during the exercise test. In addition, heart rate, blood pressure, three-lead ECG, arterial oxygen saturation and rating of perceived exertion (modified Borg scale) are measured.^31^ After the exercise test is stopped, participants continue to pedal for a 5 min recovery period, during which the work intensity is reduced to 20 W. During this recovery period, monitoring of heart rate, blood pressure, ECG, oxygen consumption and carbon dioxide production is continued.

The site investigator at each participating CPET centre determines VO_2_ peak and AT using full-page graphs of the plotted local CPET data. The VO_2_ peak is defined as the average oxygen consumption during the last 20 s of the incremental phase of exercise before attaining the limit of tolerance.^32^ The AT is determined using the modified V-Slope method.^33^ If the AT is indeterminate based on this method alone, the ventilatory equivalent method and excess carbon dioxide method are applied sequentially until the AT is either measured or classified as indeterminate.^33^ Participants, clinicians and outcome adjudicators are blinded to all CPET results, except if myocardial ischaemia or significant new arrhythmias occur during exercise, or spirometry shows previously undiagnosed very severe obstructive lung disease (forced expiratory volume in 1 s less than 30% predicted). In these cases, clinicians are informed of these specific findings, but not the VO_2_ peak or AT values.

### Other estimates of preoperative functional capacity

Each participant undergoes three other assessments of preoperative functional capacity. Subjective assessment of the participant's functional capacity is performed either by the attending doctor in the preoperative assessment clinic on the date of recruitment, or by the attending anaesthesiologist on the day of surgery. This estimate is categorised as poor (less than 4 metabolic equivalents), moderate (4–10 metabolic equivalents) or good (more than 10 metabolic equivalents). In addition, the DASI questionnaire is completed on the day of recruitment. At any point between study recruitment and initiation of surgery, a blood sample is drawn to measure the serum concentration of NT pro-BNP. These samples are initially stored at −70°C to −80°C in each study site, and then sent for analysis at the core study laboratory, the Clinical Biochemistry Laboratory at the Aberdeen Royal Infirmary (Aberdeen, UK). The NT pro-BNP samples are analysed in batches using the Siemens Vista immunoassay analyser (Siemens Healthcare Diagnostics Ltd, Frimley, UK). Clinicians and outcome adjudicators are blinded to DASI and NT pro-BNP results, while participants are blinded to NT pro-BNP results.

### Follow-up procedures

Research personnel follow the study participants daily throughout their hospital stay. While participants remain in hospital, follow-up procedures includes performance of ECGs, the Postoperative Morbidity Survey^34^ ^35^ and blood sampling to measure troponin and creatinine concentrations. The ECGs and blood sampling are performed daily for the first 3 days after surgery, while the Postoperative Morbidity Survey is administered on the third and fifth days after surgery. The specific troponin assays used are the preferred assays at each participating site. After hospital discharge, participants are contacted again at 30 days and 1 year after surgery to ascertain study-related outcomes, including vital status and health utilities measured by the EuroQol EQ-5D.^36^

### Outcome measures

The primary outcome is all-cause death or non-fatal MI within 30 days after surgery. All potential MI events are centrally adjudicated based on consensus-based definitions (table 3) by an Outcome Adjudication Committee that is blinded to all CPET, DASI and NT pro-BNP results.^37^ The secondary outcome is all-cause death within 1 year after surgery. Postoperative follow-up also includes ascertainment of other clinical events (table 3) to help further explain any differing survival associated with preoperative functional capacity.

**Table 3:** Definitions of outcomes and postoperative events

Outcome	Definition
Myocardial infarction^37^	An elevation in serum troponin that both Exceeds the 99th centile of the normal reference population Exceeds the threshold at which the coefficient of variation for the assay is 10% At least one of the following must be present: Clinical symptoms of ischaemia Typical ECG changes of ischaemia New pathological Q-waves on ECG Coronary artery intervention New (or presumed new) changes on echocardiography or radionuclide imaging
Myocardial injury^1^	An elevation in serum troponin that both Exceeds the 99th centile of the normal reference population Exceeds the threshold at which the coefficient of variation for the assay is 10%
Non-fatal cardiac arrest^1^	Successful resuscitation from documented (or presumed) ventricular fibrillation, sustained ventricular tachycardia, asystole, or pulseless electrical activity
Heart failure^1^	Presence of both Clinical findings (ie, elevated jugular venous pressure, respiratory rales, crepitations, S3 heart sounds) Radiological findings (ie, vascular redistribution, interstitial or frank pulmonary oedema)
Stroke^1^	New focal neurological deficit, suspected to vascular in origin, with signs/symptoms lasting ≥24 h
Transient ischaemic attack	Transient focal neurological deficit that lasts less than 24 h and is thought to be vascular in origin
Respiratory failure^60^	Need for tracheal intubation and mechanical ventilation after patient has completed surgery, been successful extubated, and breathing spontaneously for >1 h
Pneumonia^1^	Documented hypoxaemia (PaO_2_/FiO_2_ ratio ≤250 mm Hg) or fever (temperature >37.5°C) with either: Rales or dullness to percussion on chest examination and any of (i) new onset of purulent sputum or change in sputum character; (ii) organism isolated from blood culture; or (iii) pathogen isolated from transtracheal aspirate, bronchial brushing or biopsy New or progressive infiltrate, consolidation, cavitation or pleural effusion on chest radiograph and any of (1) criteria i, ii or iii above; (2) detection of virus or viral antigen in respiratory secretions; (3) diagnostic antibody titres; or (4) histopathological evidence of pneumonia
Surgical site infection	Physician diagnosis of surgical site infection during: Index hospitalisation Outpatient visit, hospital readmission or emergency room visit within 30 days after index surgery
Deep venous thrombosis^1^	Any of the following during index hospitalisation: Persistent intraluminal filling defect on contrast venography One or more non-compressible venous segments on B mode compression ultrasonography Clearly defined intraluminal filling defect on contrast-enhanced CT
Pulmonary embolism^1^	Any of the following during index hospitalisation: High probability ventilation/perfusion lung scan Intraluminal filling defect of segmental or larger artery on a helical CT scan Intraluminal filling defect on pulmonary angiography A positive diagnostic test for DVT (eg, positive compression ultrasound) plus low or intermediate probability ventilation/perfusion lung scan, or non-diagnostic (subsegmental defects or technically inadequate study) helical CT scan
Significant bleeding	Blood loss with any of the following characteristics: Results in drop in haemoglobin of 30 g/L or more Leads to red cell transfusion or re-operation Is considered to the cause of death
Postoperative complications*	Severity of complications are classified (based on most severe events during the index hospitalisation) as: None Mild: only temporary harm that does not require clinical treatment Moderate: required clinical treatment but without significantly prolonged hospital stay. Does not usually result in permanent harm and where this does occur, the harm does not cause functional limitation Severe—requires clinical treatment and results in significant prolongation of hospital stay and/or permanent functional limitation Fatal—death from the complication
General health utilities^36^	Measured at study recruitment, 30 days after surgery and 1 year after surgery using the EuroQol EQ-5D

*Severity of complications are classified based on scheme adapted from Clavien-Dindo classification system.^61^

DVT, deep vein thrombosis; FiO_2_, fractional inspired oxygen; PaO_2_, arterial oxygen tension.

### Statistical analysis

Since the METS Study compares several tests for predicting postoperative risk, the main statistical analyses will only include individuals who undergo their planned surgeries. Nonetheless, characteristics and outcomes of individuals who do not undergo their planned surgeries will still be captured and described separately. Two complementary analyses are planned to account for participants who are not able to exercise enough to provide a valid measurement of VO_2_ peak. Analyses will be performed only after completion of 1-year follow-up for all recruited participants.

The *primary* analysis includes individuals who successfully complete CPET by reaching their limit of tolerance with a valid measurement of VO_2_ peak. Two sets of logistic regression models will be used to separately model the risks of (1) 30-day non-fatal MI or death and (2) 1-year death. We will first include only baseline clinical data (ie, risk factors in the Revised Cardiac Risk Index),^38^ and then, in sequential fashion, add in subjective assessment, followed by VO_2_ peak to the model. The statistical significance of prognostic information from the additional predictors will be assessed based on the increase in log likelihood of the ‘larger’ model. We will also determine the area under the receiver-operating-characteristic (ROC) curve of models with successively more predictors, as well as models with only the individual exposure of interest (eg, subjective assessment alone, or VO_2_ peak alone).^39^ The difference in overall prognostic information between models will be assessed by comparing the area under the curve (AUC) of two ROC curves.^40^ We have based our sample size calculation on the AUC approach because it is commonly used in prognostic studies, and requires less speculative parameter estimates than other methods. Nonetheless, the test based on improvement in AUC may be relatively insensitive,^41^ with other methods offering more statistical power. We have therefore opted for a more conservative sample size calculation, but will use additional statistical approaches, including the logistic regression likelihood test and net reclassification improvement statistic,^42^ for further significance testing. These same methods will also be used to evaluate the additional prognostic information conveyed by DASI or NT pro-BNP.

The *secondary* analysis will include all participants who attempted CPET, regardless of whether a valid measurement of VO_2_ peak was obtained. For this analysis, CPET results will be categorised as (1) early termination for safety reasons, (2) early termination for non-cardiopulmonary reasons and (3) strata defined by the optimal VO_2_ peak cut-off points defined in the primary analysis. The same analytic approaches used in the primary analysis will then be repeated while instead expressing the results of CPET based on these categories.

### Sample size calculation

The sample size calculation is based on comparing the AUC of ROC curves for CPET versus subjective assessment with respect to predicting 30-day non-fatal MI or death.^39^ ^40^ Assuming an outcome event rate of 8%, a poor-to-moderate AUC of 0.65 for subjective assessment,^11^ ^43^ a moderately good AUC of 0.75 for VO_2_ peak,^43^ and a conservative estimated correlation of 0.5 between VO_2_ peak and subjective assessment,^13^ ^22^ a sample size of 1180 participants has 90% power to detect this clinically relevant difference in AUC values (two-sided α of 0.05). If the outcome event rate is instead 6%, this sample size has 81% power to detect the same difference. Based on studies that conducted systematic postoperative surveillance of intermediate-to-high risk patients undergoing non-cardiac surgery,^1^ ^44^ ^45^ we anticipate the rate of 30-day non-fatal MI or death to be 6–9%. This sample size of 1180 applies to the primary analysis, which is restricted to individuals who undergo their planned non-cardiac surgery and complete CPET with a valid measurement of VO_2_ peak. Thus, this analysis does not necessarily include all individuals who consent to participate in the METS Study. For example, it does not include individuals who cannot exercise sufficiently for a valid measurement of VO_2_ peak, or fail to attend their CPET session due to unexpected rescheduling of planned surgeries. To account for up to 10% of recruited participants not being eligible for inclusion in the primary analysis, the overall sample size was increased to 1312.

After recruiting half of the original planned sample size, this sample size calculation was re-evaluated based on two factors identified in the accumulating study data. *First*, we found that about 20% of participants did not either successfully complete CPET or undergo their planned surgeries. *Second*, the event rate for the primary outcome was approximately 5%. Based on this information, the overall sample size was increased to 1723 participants to account for up to 20% of recruited individuals not being eligible for the primary analysis, and a primary outcome event rate of 5%, while retaining the power of 80%. Importantly, no data on the principal exposures (ie, CPET results, DASI scores, NT pro-BNP concentration) were considered during this sample size re-estimation.

### Study management and funding

The Applied Health Research Centre at St Michael's Hospital (Toronto, Ontario, Canada) is responsible for the overall international coordination of the METS Study. Two national coordinating centres also help liaise with local investigators in specific countries, namely the Royal London Hospital (London, UK) for the UK, and the Alfred Hospital (Melbourne, Victoria, Australia) for Australia and New Zealand. The study investigators participating in the METS Study, as well as their respective roles, are listed in the online supplementary data appendix. All study data are captured with electronic case record forms on a secure web-based database that was developed using Medidata RAVE (Medidata Solutions Inc, New York, New York, USA). The METS Study is funded by peer-reviewed grants from the Canadian Institutes of Health Research, Heart and Stroke Foundation of Canada, Ontario Ministry of Health and Long-Term Care, National Institute of Academic Anaesthesia, UK Clinical Research Network, Australian and New Zealand College of Anaesthetists, and Monash University (Melbourne, Victoria, Australia).

10.1136/bmjopen-2015-010359.supp1 Supplementary data

### Study status

Participant recruitment to the METS Study was started in March 2013. The study involves 25 participating centres in Australia, Canada, New Zealand and the UK. Completion of 1-year follow-up period is anticipated for late 2016.

### Substudies

We have developed a formal process for investigators within the research group to propose, design and lead substudies based on the data collected from this large international cohort of patients undergoing major elective non-cardiac surgery. Three substudies have already been prespecified. The first substudy will evaluate the prognostic accuracy of AT as determined by site investigators at each participating CPET centre. The second substudy will evaluate the prognostic accuracy of VO_2_ peak and AT measurements that are centrally adjudicated by a panel of three CPET experts. These experts will remain blinded to initial assessments made by the local site investigators at each CPET centre. The third substudy will investigate the role of the 6 min walk test (6MWT) for assessing preoperative functional capacity and predicting postoperative outcome.^46^ This simple and inexpensive exercise test may help stratify surgical patients based on their performance on CPET.^47^ In a subset of study participants, we will assess the ability of the 6MWT to predict short-term postoperative quality of recovery,^48^ medium-to-long term disability after surgery,^49^ and performance on CPET.

## Ethics and dissemination

The METS Study has received research ethics board approval at all participating sites. The study poses minimal additional risk to study participants. Specifically, all CPET assessments are performed under close medical supervision. In addition, prior data show CPET to be very safe, with major complications occurring in 8–13 per 100 000 tests, and death in 2–5 per 100 000 tests.^30^ It has an established role for assessing patients with cardiopulmonary disease,^30^ and can be performed safely in high-risk populations, such as individuals with pulmonary hypertension or small abdominal aortic aneurysms.^50^ ^51^ While the primary results (ie, VO_2_ peak and AT) of each CPET assessment remain concealed until completion of the study, clinicians responsible for study participants are informed of other specific high-risk findings during exercise testing, such as myocardial ischaemia or significant new arrhythmias.

The results of the METS Study will be published in peer-reviewed journals, in addition to being presented at national and international conferences. We anticipate these results to be published in 2017, after completion of 1-year follow-up of all recruited participants. We will also liaise with representatives of relevant clinical practice guideline organisations to ensure that the study findings will help inform future recommendations for perioperative care.^3^ ^4^

## Conclusions

By defining the most accurate approaches for evaluating preoperative cardiopulmonary fitness, the results of the METS Study will help clinicians to better identify high-risk patients who would benefit from preoperative optimisation, interventions, haemodynamic management, closer postoperative surveillance or avoidance of surgery. Furthermore, once patients with poor functional capacity can be more accurately identified, opportunities will arise for randomised controlled trials of interventions to improve their outcomes, such as preoperative exercise training programmes,^52^ perioperative haemodynamic optimisation^53^ ^54^ and enhanced postoperative care (eg, hospitalist-surgeon co-management models).^55–57^ Thus, the METS Study has the potential to substantially inform and improve the care of the millions of individuals who undergo major surgery worldwide every year.^2^
