# Supplementary material for: Measurement of Exercise Tolerance before Surgery (METS) study: a protocol for an international multicentre prospective cohort study of cardiopulmonary exercise testing prior to major non-cardiac surgery
Source: BMJ Open. 2016 Mar 11;6(3):e010359. doi: 10.1136/bmjopen-2015-010359 (PMC4800144; doi:10.1136/bmjopen-2015-010359)
Supplement: Supplementary data [file bmjopen-2015-010359supp.pdf]

SUPPLEMENTARY DOCUMENTS

1. **Study Site Investigators**

Australia – *Alfred Hospital*: P S Myles (Site Co-Lead), M A Shulman (Site Co-Lead), S Wallace, C Farrington, B Thompson (Site CPET Lead), M Ellis, B Borg; *John Hunter Hospital*: R Kerridge (Site Lead), J Douglas, J Brannan, J Pretto; *Nambour General Hospital*: M G Godsall (Site Co-Lead), N Beauchamp (Site Co-Lead), S Allen, A Kennedy, E Wright, J Malherbe; *Peter McCallum Cancer Centre*: H Ismail (Site Lead), B Riedel, A Melville, H Sivakumar, A Murmane, K Kenchington; *Prince Charles Hospital*: U Gurunathan (Site Lead), C Stonell, K Brunello, K Steele, O Tronstand, P Masel, A Dent, E Smith, A Bodger, M Abolfathi; *Princess Alexandra Hospital*: P Sivalingam (Site Co-Lead), A Hall (Site Co-Lead); *Royal Adelaide Hospital*: T Painter (Site Lead), A Elliott, A M Carrera; *Royal Hobart Hospital*: N C S Terblanche (Site Lead); S Pitt, J Samuels, C Wilde; *Royal Melbourne Hospital*: M MacCormick (Site Lead), K Leslie; *Western Health*: D Bramley (Site Lead), A M Southcott, J Grant, H Taylor, S Bates, M Towns, A Tippett, F Marshall

Canada – *St Michael's Hospital*: C D Mazer (Site Lead), J Kunasingam, A Yagnik, C Crescini; *Sunnybrook Health Sciences Centre*: C J L McCartney (Site Co-Lead), S Choi (Site Co-Lead), P Somascanthan, K Flores; *Toronto General Hospital*: D N Wijeysundera (Site Co-Lead), W S Beattie (Site Co-Lead), K Karkouti, H A Clarke, A Jerath, S A McCluskey, M Wasowicz, J T Granton (Site CPET Lead), L Day, J Pazmino-Canizares; *Toronto Rehabilitation Institute – Rumsey Centre*: P Oh (Site Lead), R Belliard, L Lee, K Dobson; *Toronto Western Hospital*: V Chan (Site Co-Lead), R Brull (Site Co-Lead), N Ami, M Stanbrook (Site CPET Lead)

New Zealand - *Auckland City Hospital*: K Kagen (Site Lead), D Campbell, T Short, J Van Der Westhuizen, K Higgie, H Lindsay, R Jang, C Wong, D Mcallister, M Ali, J Kumar, E Waymouth; *Middlemore Hospital*: J Dimech (Site Co-Lead), M Lorimer (Site Co-Lead), R Sara, A Collingwood, S Olliff, S Gabriel, H Houston; *Wellington Hospital*: P Dalley (Site Lead), S Hurford, A Hunt, L Andrews, L Navarra, A Jason-Smith

United Kingdom – *Aberdeen Royal Infirmary*: B L Croal (Site Lead), M Lum; *Royal Free Hospital*: D Martin (Site Lead), S James; *Royal London Hospital*: R M Pearse (Site Lead), T E F Abbott, M Phull, C Beilstein, P Bodger, K Everingham, Y Hu, E Niebrzegowska, C Corriea, T Creary, M Januszekska, T Ahmad, J Whalley, R Haslop, J McNeil, A Brown, N MacDonald; *Royal Marsden Hospital*: S Jhani (Site Co-Lead), R Raobaikady (Site Co-Lead), E Black, M Rooms, H Lawrence; *Southampton General Hospital*: S Jack (Site Co-Lead), M Celinski (Site Co-Lead), D Levett, M Edwards, K Salmon, C Bolger, L Loughney, L Seaward, H Collins, B Tyrell, N Tantony, K Golder; *University College London Hospital*: G Ackland (Site Lead), R C M Stephens, L Gagello-Paredes; *Whipps Cross Hospital*: A Raj (Site Lead), R Lifford

2. **National Coordinators** – *Australia and New Zealand*: P S Myles, M A Shulman; *Canada*: D N Wijeysundera; *United Kingdom*: R M Pearse, T E F Abbott
3. **Project Office Operations Committee** – B H Cuthbertson, D N Wijeysundera, E Torres, M Melo, M Mamdani, K E Thorpe, R M Pearse, T E F Abbott, P S Myles, M A Shulman, S Wallace, C Farrington, B L Croal
4. **CPET Methods Committee** – M P W Grocott, J T Granton, P Oh, B Thompson, D Levett
5. **Outcome Adjudication Committee** – G Hillis (Chair), W S Beattie, H C Wijeysundera

6. **International Steering Committee –** B H Cuthbertson (International Co-Principal Investigator), D N Wijeysundera (International Co-Principal Investigator), R M Pearse, M A Shulman, T E F Abbott, E Torres, B L Croal, J T Granton, K E Thorpe, M P W Grocott, C Farrington, S Wallace, P S Myles
